# Performance of the ARC-HBR criteria in ST-elevation myocardial infarction. Significance of smoking as an additional bleeding risk factor

**DOI:** 10.1093/ehjqcco/qcae104

**Published:** 2024-11-30

**Authors:** Henri Kesti, Kalle Mattila, Samuli Jaakkola, Joonas Lehto, Nea Söderblom, Kalle Kalliovalkama, Pekka Porela

**Affiliations:** Heart Centre, Turku University Hospital and University of Turku, Hämeentie 11, PO Box 52, 20521 Turku, Finland; Faculty of Medicine, University of Turku, Kiinamyllynkatu 10, 20520 Turku, Finland; Faculty of Medicine, University of Turku, Kiinamyllynkatu 10, 20520 Turku, Finland; Emergency Department, Turku University Hospital, Hämeentie 11, PO Box 52, 20521 Turku, Finland; Heart Centre, Turku University Hospital and University of Turku, Hämeentie 11, PO Box 52, 20521 Turku, Finland; Heart Centre, Turku University Hospital and University of Turku, Hämeentie 11, PO Box 52, 20521 Turku, Finland; Heart Centre, Turku University Hospital and University of Turku, Hämeentie 11, PO Box 52, 20521 Turku, Finland; Faculty of Medicine, University of Turku, Kiinamyllynkatu 10, 20520 Turku, Finland; Heart Centre, Turku University Hospital and University of Turku, Hämeentie 11, PO Box 52, 20521 Turku, Finland; Faculty of Medicine, University of Turku, Kiinamyllynkatu 10, 20520 Turku, Finland; Heart Centre, Turku University Hospital and University of Turku, Hämeentie 11, PO Box 52, 20521 Turku, Finland

**Keywords:** ST-elevation myocardial infarction, STEMI, Acute coronary syndrome, Smoking, Bleeding, Academic research consortium

## Abstract

**Background and aims:**

The Academic Research Consortium for High Bleeding Risk criteria (ARC-HBR) are recommended by guidelines for bleeding risk assessment in ST-elevation myocardial infarction (STEMI). The aim of this study was to identify possible other risk factors and adjust the original ARC-HBR criteria for confounders.

**Methods and results:**

All consecutive STEMI patients managed in a Finnish tertiary hospital between 2016 and 2022 were identified using a database search. Data collection was done by reviewing electronic patient records. Bleeding risk was assessed according to the ARC-HBR criteria. The primary endpoint was non-access site bleeding academic research consortium (BARC) type 3 or 5 bleeding during 1-year follow-up. A total of 1548 STEMI patients were analysed. HBR criteria was fulfilled in 661 (42.7%). Multivariable competing risk analysis identified only 4 individual ARC-HBR criteria as independent risk factors for bleeding. Smoking status was identified as a novel bleeding risk factor. Current and former smokers had increased bleeding risk compared with never smokers [hazard ratio (HR) 3.01, 95% confidence interval (CI) 1.62–5.61 and HR 1.99, CI 1.19–3.34]. In those not meeting any ARC-HBR criteria, cumulative BARC 3 or 5 incidence of current smoking was 3.40% and intracranial haemorrhage (ICH) 1.36%. Thus, exceeding ARC-HBR definition for a major criterion. In the non-HBR group the prevalence of current smoking was 40.4% (*n* = 358).

**Conclusion:**

Current and former smoking predicts major bleeding complications after STEMI. Current smoking is highly prevalent among those classified as non-HBR according to the ARC-HBR criteria.

## Graphical Abstract

**Graphical Abstract:**
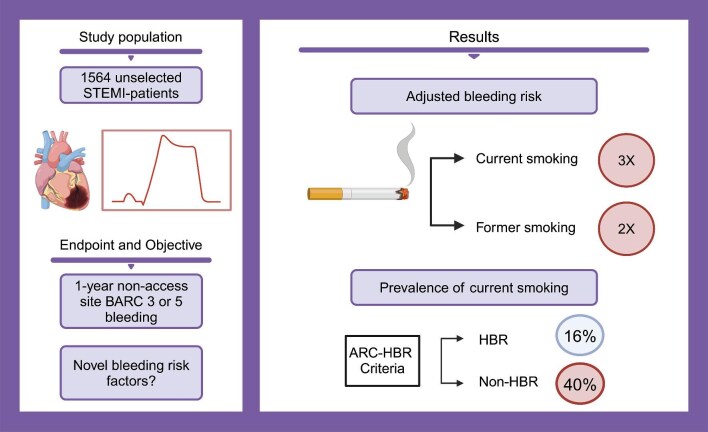
Created in BioRender.

Key learning points **What is already known**

- The Academic Research Consortium for High Bleeding Risk Criteria (ARC-HBR) have successfully predicted increased bleeding risk after an acute coronary syndrome but variability in the performance of individual criteria exists.
- Significance of bleeding risk factors seems to differ between subcategories of acute coronary syndrome and stable coronary artery disease.
- Possible other bleeding risk factors outside the ARC-HBR criteria are rarely considered and patients with STEMI are often underrepresented in previous studies.

**What this study adds**

- After adjustment for confounders, from the ARC-HBR criteria age ≥75 years, active malignancy, chronic kidney disease, and anaemia were independent predictors of major bleeding at 1-year. Chronic kidney disease and anaemia seem to be consistently associated with increased bleeding risk.
- From other variables, increase in white blood cell count was associated with a modest increase in bleeding risk. Notably, compared with never smoking, current smoking increased bleeding risk three-fold and former smoking two-fold.
- Over 40% of patients classified as not high bleeding risk according to the ARC-HBR criteria were current smokers. Thus, recognizing smoking as a bleeding risk factor could help to identify a large proportion of high bleeding risk patients who would otherwise be missed.

## Introduction

Identification of high bleeding risk (HBR) patients is essential in the management of acute coronary syndromes (ACS), as major bleeding complications are associated with increased mortality.^1^ Following percutaneous coronary intervention (PCI), HBR patients benefit from shortened dual antiplatelet therapy (DAPT) treatment durations due to reduction in bleeding complications.^2^ Therefore, identifying those at HBR is important when making treatment decisions on patients with ACS. Academic Research Consortium for High Bleeding Risk (ARC-HBR) defined a set of clinical parameters to identify HBR patients.^3^ European guidelines recommend using these criteria to evaluate bleeding risk in patients with ST-elevation myocardial infarction (STEMI).^4^ Among patients with ACS, those with STEMI seem to have the highest bleeding risk.^5^ Furthermore, STEMI patients are generally underrepresented in randomized DAPT trials and registry-based studies investigating bleeding risk. Therefore, additional studies on bleeding risk in this population are needed.

According to the ARC-HBR consensus, HBR was defined as 1-year bleeding academic research consortium (BARC) type 3 or 5 bleeding risk ≥4% or intracranial haemorrhage (ICH) risk ≥1%. Only a few studies have investigated how the ARC-HBR criteria perform when adjusted for other possible confounding bleeding risk factors. This might be due to the assumption that the criteria already include all relevant clinical characteristics associated with bleeding.^6,7^ After adjustment for confounders, only few individual ARC-HBR criteria have remained associated with increased bleeding risk.^8,9^ After the first establishment of the criteria in 2018, new bleeding risk factors such as clinical presentation with ACS vs. chronic coronary syndrome (CCS) have been identified.^10^ For other variables such as smoking status, evidence regarding association with bleeding remains contradictory.^10,11^

We hypothesized there are significant major bleeding predictors not included in the ARC-HBR criteria among STEMI patients. Therefore, the aim of this study was to identify these predictors and adjust the original ARC-HBR criteria for confounders to evaluate which criteria are significant among STEMI.

## Methods

### Data sources

Data used in this study was gathered from the institutional database of The Hospital District of Southwest Finland (VSSHP). VSSHP is a public municipal authority that produces specialized health care services to the 480 000 residents of the region. All STEMI patients from the region are treated in Turku University Hospital, which has round-the-clock primary PCI capability. VSSHP institutional database contains information stored in electronic patient information systems. Electronic patient records in the VSSHP patient information system were inspected with a standardized data collection protocol. Data on the date and cause of death were obtained from Statistics Finland, a governmental agency which reviews all death certificates issued in Finland.

### Study population

A database search of the VSSHP institutional database was conducted by Auria Clinical Informatics. The database was searched for hospitalizations between 2016 and 2022 for STEMI according to the International Classification of Diseases, 10th revision (ICD-10) codes I21.0-I21.3. For patients admitted more than once during the study period, only the first admission was included. All consecutive adults presenting with STEMI and residing in VSSHP catchment areas at the time of index hospitalization were included to ensure reliable follow-up data. STEMI diagnosis was confirmed by study personnel using electronic patient records. In case of uncertainty regarding diagnosis, the final decision was made by an independent interventional cardiologist (AY). STEMI was defined according to the fourth universal definition of myocardial infarction (MI).^12^

### Baseline characteristics

Baseline laboratory values were acquired from the database search. The authors had access to all existing patient records from VSSHP. Medication, smoking status, alcohol consumption, left ventricular ejection fraction (LVEF), comorbidities, prior cardiac procedures, management, and prescribed duration of DAPT were gathered from patient records.

Smoking status was evaluated by reviewing records during index-hospitalization and follow-up. Text searches from all existing patient records at any time were also conducted with appropriate phrases. Current smoking was defined as actively smoking at the time of index STEMI and former smoking as history of smoking, but the patient had quit before index event. Patients were considered never smoking if it was directly mentioned in patient records or smoking was not mentioned at all in the records. In a sensitivity analysis, those who had no mention of smoking were excluded. Alcohol consumption was evaluated by reviewing patient records and text search results. Excessive alcohol consumption was defined as any of the following within 12 months of index-hospitalization: alcohol-related diagnosis, hospitalization or emergency department visit due to excessive alcohol use, excessive consumption mentioned in patient records, alcohol doses per week >22 for men and >11 for women. Not mentioned was considered non-excessive consumption. In a separate sensitivity analysis, those who had no mention of alcohol were excluded. LVEF was searched from echocardiography reports during index-hospitalization. If visual inspection and measured LVEF were used, the measured one was preferred.

Measured low-density lipoprotein >3 mmol/L was considered hypercholesterolaemia even if the diagnosis was not made by treating physician. New atrial fibrillation was included if it was not considered momentarily caused by myocardial ischaemia. Delayed PCI was defined as PCI > 24 h after symptom onset. Only the first revascularization procedure was recorded. Medication was assessed at discharge or at the time of endpoint event or death if they occurred during index-hospitalization.

### Bleeding risk assessment

Bleeding risk was assessed according to the ARC-HBR criteria, which have been described previously.^3^ Patients were considered at HBR if at least 1 major or 2 minor criteria were met. Estimated glomerular filtration rate (eGFR) was calculated using Chronic Kidney Disease Epidemiology Collaboration equation. In the present study, mild anaemia was defined as haemoglobin 110–129 g/L for men and 110–119 g/L for women (ARC-HBR minor criterion) and moderate or severe anaemia as haemoglobin <110 g/L (major criterion). Moderate CKD was defined as eGFR 30–59.99 mL/min (ARC-HBR minor criterion) and severe or end-stage CKD as eGFR <30 mL/min (major criterion). The major criterion for prior stroke was slightly modified and defined as follows: previous spontaneous ICH at any time or traumatic ICH within the past 12 months; presence of brain arteriovenous malformation; ischaemic stroke within the past 6 months defined as sudden onset of neurological signs or symptoms fitting a focal or multifocal vascular territory within the brain, spinal cord, or retina, that persist for ≥24 h or until death and confirmed by neuroimaging or if no imaging was performed, the diagnosis was set by treating neurologist.

### Endpoints and follow-up

The primary endpoint was a composite of non-access site BARC type 3 or 5 bleeding during 1-year follow-up.^13^ We chose to exclude access site bleeding events due to their lesser prognostic significance.^14,15^ Detailed endpoint definitions are provided in Supplementary *Table S1*. Patients were followed starting from the hospitalization date until first occurrence of endpoint or 365 days. All existing electronic patient records of the study subjects that were available in the VSSHP patient information system during the follow-up period were inspected for bleeding events. Additionally, causes of death were inspected to identify possible fatal bleeding events occurring outside healthcare facilities.

### Statistical analyses

Statistical analyses were conducted with R statistics software (Version 4.3.0, R Foundation for Statistical Computing, Vienna Austria) and SPSS Statistics (Version 27.0.1.0). A *P*-value < 0.05 was considered significant and significance analyses were two-tailed. Categorical variables are presented as frequencies (and percentages) and were compared using Chi-square test or Fisher's exact test as appropriate. Continuous variables are presented as mean (SD) or median (IQR) and compared using *t*-test or Mann–Whitney U test as appropriate. Normality was assessed by visual inspection of histograms, computation of Q–Q plots and using skewness and kurtosis. Univariable and multivariable competing risk analysis with the Fine–Gray subdistribution hazard model with non-bleeding-related death as a competing event was used to estimate the association between baseline characteristics and bleeding. This method was chosen because death is a significant competing event after STEMI that modifies the chance that bleeding occurs during follow-up. The proportional hazards assumption was assessed using Schoenfeld residuals.

The prespecified multivariable model included all individual ARC-HBR criteria with *P* < 0.05 in univariable analysis and variables not included in the criteria that remained significant (*P* < 0.05) in univariable analysis. If both major and minor ARC-HBR criterion of the same variable (for example anaemia) had *P* < 0.05 in univariable analysis, major criterion was chosen. If significant variables were abundant and the model faced overfitting (more than 1 variable in model per 10 events), variables to be included were chosen based on existing evidence and clinical relevance. Exact bleeding sites other than ICH of newly identified independent bleeding predictors were inspected in post-hoc analyses.

### Research permits and ethical considerations

This study was approved by the Institutional Review Board of VSSHP. A license to access causes of death was granted by Statistics Finland. Informed consent and ethical board review were waived due to the study design. According to the Finnish Medical Research Act (488/1999) ethical review is not required for retrospective studies. The legal basis for processing personal data in this study is public interest and scientific research, EU General Data Protection Regulation 2016/679 (GDPR), Article 6(1)(e) and Article 9(2)(j).

Prior to publication the results were inspected for anonymity by Findata, which is the data permit authority for the social and health care sector in Finland. Its activities are based on Finnish Act on the Secondary Use of Health and Social Data (522/2019). Based on their review, some frequencies and equivalent percentages with values <3 had to be censored to ensure anonymity of the study subjects. These were reported as symbol ‘<’ followed by the smallest possible value that was considered anonymized. Censoring did not affect data gathering or the results. Statistical analyses were conducted using the original data values.

## Results

### Patient population

The formation of the study population is shown in Supplementary *Figure S1*. Overall, 661 (42.7%) were HBR and 887 (57.3%) were non-HBR. The prevalence of individual ARC-HBR criteria is summarized in Supplementary *Figure S2*. Most fulfilled minor criteria were age ≥75 years (*n* = 543, 34.7%), moderate CKD (*n* = 323, 20.7%), and mild anaemia (*n* = 272, 17.4%). Most frequent major criteria were oral anticoagulation (OAC) (*n* = 219, 14.0%), moderate or severe anaemia (*n* = 128, 8.18%), severe or end-stage CKD (*n* = 72, 4.60%), bleeding diathesis (*n* = 67, 4.28%), and active malignancy (*n* = 60, 3.82%). The amount of missing data was low and is reported in Supplementary *Table S3*.

### Clinical characteristics

Clinical characteristics, medication and management are summarized in *Tables 1* and *2*. Patients at HBR were older, had more comorbidities and prior coronary procedures. Non-HBR patients were more often current smokers compared with HBR patients (40.4% vs. 16.6%, *P* < 0.001).

**Table 1:** Clinical characteristics and laboratory values

Variable	Overall (*n* = 1564)	HBR (*n* = 661)	Non-HBR (*n* = 887)	*P*-value
Clinical characteristics				
Sex				
Female, *n* (%)	478 (30.6)	262 (39.6)	209 (23.6)	<0.001
Male, *n* (%)	1086 (69.4)	399 (60.4)	678 (76.4)	
Age, median (Q1, Q3)	69.6 (58.7, 76.4)	76.9 (69.2, 83.7)	63.7 (55.3, 70.4)	<0.001
Smoking				
Current, *n* (%)	473 (30.2)	110 (16.6)	358 (40.4)	<0.001
Former, *n* (%)	356 (22.8)	181 (27.4)	173 (19.5)	
Never, *n* (%)	735 (47.0)	370 (56.0)	356 (40.1)	
Alcohol consumption				
Excessive, *n* (%)	118 (7.5)	46 (7.0)	72 (8.1)	0.396
Non-excessive, *n* (%)	1430 (91.4)	615 (93.0)	815 (91.9)	
LVEF <35, *n* (%)	242 (15.5)	137 (20.7)	97 (10.9)	<0.001
Hypertension, *n* (%)	882 (56.4)	451 (68.2)	424 (47.8)	<0.001
Hypercholesterolaemia, *n* (%)	914 (58.4)	346 (52.3)	564 (63.6)	<0.001
Diabetes, *n* (%)	350 (22.4)	172 (26.0)	174 (19.6)	0.003
Atrial fibrillation, *n* (%)	229 (14.6)	218 (33.0)	10 (1.1)	<0.001
Heart failure, *n* (%)	91 (5.8)	76 (11.5)	14 (1.6)	<0.001
Prior CAD, *n* (%)	657 (42.0)	317 (48.0)	331 (37.3)	<0.001
Prior MI, *n* (%)	215 (13.7)	124 (18.8)	88 (9.9)	<0.001
Prior PCI, *n* (%)	192 (12.3)	101 (15.3)	89 (10.0)	0.002
Prior CABG, *n* (%)	45 (2.9)	34 (5.1)	10 (1.1)	<0.001
Peripheral artery disease, *n* (%)	59 (3.8)	43 (6.5)	15 (1.7)	<0.001
Prior stroke, *n* (%)	111 (7.1)	97 (14.7)	12 (1.4)	<0.001
Prior ICH, *n* (%)	36 (2.3)	32 (4.8)	3 (0.3)	<0.001
Laboratory values				
Haemoglobin, g/L, mean (SD)	135.8 (18.2)	126.0 (19.4)	143 (12.9)	<0.001
Thrombocytes, ×10^9^/L, median (Q1, Q3)	237 (201, 280)	227 (184, 275)	242 (209, 283)	<0.001
White blood cell count, ×10^9^/L, median (Q1, Q3)	10.1 (8.0, 12.4)	10.1 (7.6, 12.5)	10.2 (8.3, 12.3)	0.684
Creatinine, mmol/L, median (Q1, Q3)	83.0 (69.0, 96.0)	93.0 (77.0, 116)	78.0 (67.0, 88.0)	<0.001
HbA1c, mmol/mol, median (Q1, Q3)	39.0 (35.0, 43.0)	39.0 (36.0, 43.0)	38.0 (35.0, 42.0)	<0.001

HBR, high bleeding risk; LVEF, left ventricular ejection fraction; CAD, coronary artery disease; MI, myocardial infarction; PCI, percutaneous coronary intervention; CABG, coronary artery bypass grafting; and ICH, intracranial haemorrhage.

**Table 2:** Medication and management

Variable	Overall (*n* = 1564)	HBR (*n* = 661)	Non-HBR (*n* = 887)	*P*-value
Medication at discharge^#^				
ASA, *n* (%)	1374 (87.9)	496 (75.0)	872 (98.3)	<0.001
Clopidogrel, *n* (%)	410 (26.2)	306 (46.3)	103 (11.6)	<0.001
Ticagrelor, *n* (%)	964 (61.6)	217 (32.8)	744 (83.9)	<0.001
Prasugrel, *n* (%)	13 (0.8)	5 (0.8)	8 (0.9)	0.756
DAPT, *n* (%)	1274 (81.5)	422 (63.8)	849 (95.7)	<0.001
DAPT duration				
<3 months, *n* (%)	88 (6.9)*	79 (18.7)*	9 (1.1)*	<0.001
3–5.9 months, *n* (%)	39 (3.1)*	25 (5.9)*	14 (1.6)*	
6–9 months, *n* (%)	119 (9.3)*	57 (13.5)*	62 (7.3)*	
12 months, *n* (%)	1028 (80.7)*	261 (61.8)*	764 (90.0)*	
DAPT with ticagrelor or prasugrel, *n* (%)	959 (61.3)	210 (31.8)	746 (84.1)	<0.001
VKA, *n* (%)	53 (3.4)	53 (8.0)	0 (0.0)	<0.001
DOAC, *n* (%)	166 (10.6)	166 (25.1)	0 (0.0)	<0.001
TAT, *n* (%)	80 (5.1)	80 (12.1)	0 (0.0)	<0.001
NSAID, *n* (%)	<6 (<0.38)^§^	3 (0.45)	<3 (< 0.34)^§^	0.319
Corticosteroid, *n* (%)	53 (3.4)	46 (7.0)	7 (0.8)	<0.001
PPI, *n* (%)	602 (38.5)	362 (54.8)	234 (26.4)	<0.001
Management				
Primary PCI, *n* (%)	1367 (87.4)	518 (78.4)	838 (94.5)	<0.001
Delayed PCI, *n* (%)	35 (2.2)	25 (3.8)	10 (1.1)	<0.001
Fibrinolysis, *n* (%)	<6 (<0.38)^§^	<3 (< 0.45)^§^	<3 (<0.34)^§^	1.000
Rescue PCI, *n* (%)	<6 (<0.38)^§^	<3 (< 0.45)^§^	<3 (<0.34)^§^	1.000
CABG, *n* (%)	26 (1.7)	11 (1.7)	15 (1.7)	0.967
Angiography without revascularization, *n* (%)	35 (2.2)	26 (3.9)	9 (1.0)	<0.001
Non-invasive, *n* (%)	97 (6.2)	79 (12.0)	13 (1.5)	<0.001

#Or during endpoint if occurred before discharge.

*Of those with DAPT.

^§^Value <3 censored based on the review of Findata (data permit authority for the social and health care sector in Finland) to ensure anonymity of study subjects.

ASA, acetylsalicylic acid; DAPT, dual antiplatelet therapy; DAPT duration, prescribed duration of DAPT; VKA, vitamin K antagonist; DOAC, direct oral anticoagulant; TAT, triple antithrombotic therapy (ASA + P2Y12 receptor inhibitor + anticoagulant); NSAID, nonsteroidal anti-inflammatory drug; PPI, proton pump inhibitor; PCI, percutaneous coronary intervention; delayed PCI, performed >24 h after symptom onset; and CABG, coronary artery bypass grafting.

Overall, 87.4% received primary PCI and 2.2% delayed PCI. In total, 8.4% (*n* = 132) were managed without revascularization and this was more common among HBR-patients and elderly. Median age of those managed without revascularization was 83.8 years [interquartile range (IQR) 76.2–88.7] vs. 68.6 years (IQR 59.1–76.9) among revascularized patients (*P* < 0.001). Coronary artery bypass grafting, fibrinolysis, and rescue PCI were rare in both groups. HBR-patients were less often prescribed DAPT treatment and a potent P2Y12 inhibitor i.e. ticagrelor or prasugrel as compared with non-HBR patients. The prescribed duration of DAPT was generally shorter among HBR-patients. In the HBR group, only 61.8% of those on DAPT had duration of 12 months, whereas in the non-HBR group, the amount was 90.0%. Use of proton-pump inhibitors was more common among HBR patients as compared to non-HBR patients (54.8% and 26.4%, respectively).

### Clinical outcomes

**Figure 1:**
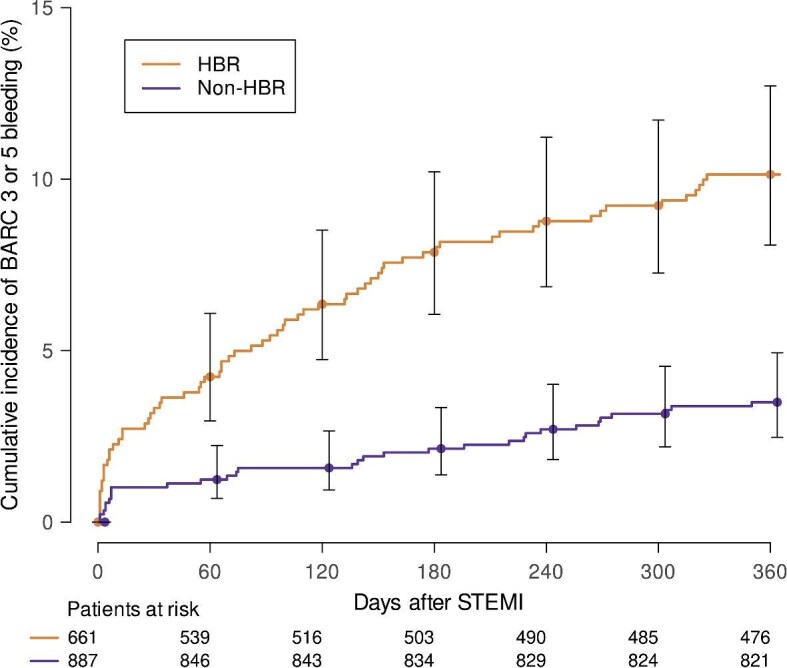
Cumulative incidence of BARC 3 or 5 bleeding according to bleeding risk status. HBR, high bleeding risk; BARC, bleeding academic research consortium; and STEMI, ST-elevation myocardial infarction.

During 1-year follow up, BARC 3 or 5 cumulative incidence was higher among those at HBR compared to non-HBR patients (10.1%, *n* = 67 vs. 3.49%, *n* = 31) (*Figure 1*). The risk of 1-year BARC 3 or 5 bleeding was three-fold higher in HBR compared to non-HBR (unadjusted hazard ratio 3.01, 95% confidence interval 1.97–4.61, *P* < 0.001). No BARC 3 or 5 events occurred among those 16 patients of which the ARC-HBR criteria were not assessable due to missing haemoglobin, thrombocytes and creatinine values. BARC subtypes are reported in Supplementary *Table S2*. Bleeding sites are summarized in Supplementary *Figure S3*.

### Adjusted performance of the ARC-HBR criteria and other bleeding risk factors

Univariable regression analyses for 1-year BARC 3 or 5 bleeding are shown in Supplementary *Table S3*. After adjustment (*Table 3*), from the ARC-HBR criteria age, CKD, anaemia, and active malignancy remained independent bleeding predictors. Smoking status was identified as a bleeding risk factor. Current smoking increased bleeding risk three-fold and former smoking two-fold compared to never smoking. Smoking remained associated with increased bleeding risk in sensitivity analyses which were performed on only those with known smoking status and only PCI-treated patients (Supplementary *Tables S4* and *S5*). Increased white blood cell count also remained independent bleeding predictor.

**Table 3:** Multivariable Fine-Gray regression model for 1-year BARC 3 or 5 bleeding

Variable	HR	95% CI	*P*-value
Age ≥75 years*	2.36	1.38–4.03	0.002
OAC*	1.47	0.75–2.86	0.260
GFR 30–59.99 mL/min*	1.63	1.02–2.61	0.040
Haemoglobin <110 g/L*	1.96	1.06–3.63	0.031
Prior bleeding (major criterion)*	1.88	0.71–4.99	0.210
Active malignancy*	3.11	1.57–6.15	0.001
Smoking	–	–	<0.001
Current^§^	3.01	1.62–5.61	<0.001
Former^#^	1.99	1.19–3.34	0.009
White blood cell count (1 × 10^9^/L)	1.03	1.00–1.07	0.031
DAPT-duration^$^	–	–	0.280
PPI	1.50	0.98–2.29	0.060

*ARC-HBR criterion

^§^Former smoking excluded (current vs. never)

^#^Current smoking excluded (former vs. never)

^$^Categories: no DAPT (reference), <3 months, 3–5.9 months, 6–9 months, 12 months. HR and CI for category comparisons not provided because the variable was not significant.

In the model: Individual significant (Univariable Fine-Gray *P* < 0.05) ARC-HBR criteria and other significant variables. If both major and minor criterion of the same variable was significant, major criterion was included.

BARC, Bleeding Academic Research Consortium; HR, hazard ratio; CI, confidence interval; OAC, oral anticoagulant; and GFR, estimated glomerular filtration rate (CKD-EPI formula); active malignancy, diagnosis within 12 months prior to index hospitalization or ongoing treatment.

### Bleeding incidence according to smoking status

**Figure 2:**
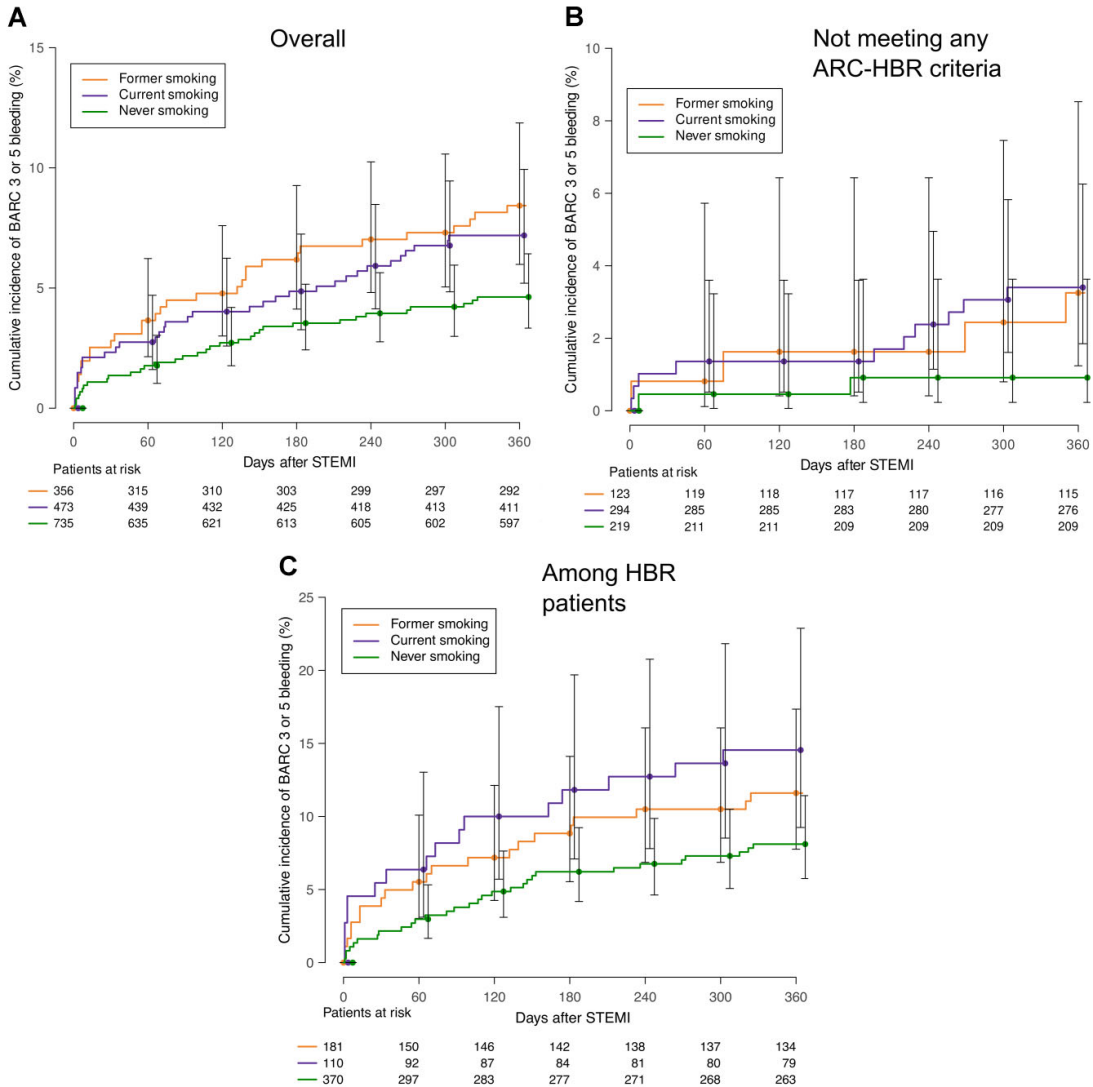
Cumulative incidence of BARC 3 or 5 bleeding according to smoking status. BARC, bleeding academic research consortium; STEMI, ST-elevation myocardial infarction; ARC-HBR, Academic Research Consortium for High Bleeding Risk; and HBR, high bleeding risk. (*A*) overall study population; (*B*) among those not meeting any ARC-HBR criteria; and (*C*) among those classified as HBR according to ARC-HBR criteria.

Cumulative incidences of 1-year BARC 3 or 5 bleeding among current, former and never smokers were 7.19%, 8.43%, and 4.63% and the cumulative 1-year ICH incidences were 1.48%, 1.12%, and 0.82%, respectively. The cumulative incidences of BARC 3 or 5 and ICH were 3.40% and 1.36%, respectively in current smokers who did not meet any ARC-HBR criteria, exceeding the ARC-HBR definition for a major criterion. BARC 3 or 5 cumulative incidences among former and never smokers who did not meet any ARC-HBR criteria were 3.25% and 0.91% and ICH incidences 0.81% and 0.0%, respectively. *Figure 2* shows BARC 3 or 5 cumulative incidence stratified by smoking status. Bleeding sites according to smoking status are shown in Supplementary *Table S6*.

### Including current smoking as a major ARC-HBR criterion

Of non-HBR patients, a total of 358 (40.4%) were current smokers. If current smoking was considered a major ARC-HBR criterion, these patients would be considered as HBR. This would result in 65.8% (*n* = 1019) of the study population belonging in the HBR-group (compared to the original 42.7%). Majority of non-HBR patients (71.7%, *n* = 636) did not meet any ARC-HBR criteria. The additive impact of current and former smoking in relation to fulfilment of the ARC-HBR criteria is demonstrated in *Table 4*.

**Table 4:** Additive impact of smoking status on 1-year BARC 3 or 5 bleeding incidence

Smoking status	No ARC-HBR criteria met	1 minor criterion met	HBR
Current	$\frac{{10}}{{294}},\ 3.4\% $	$\frac{8}{{64}},\ 12.5\% $	$\frac{{16}}{{110}},\ 14.5\% $
Former	$\frac{4}{{123}},\ 3.3\% $	$\frac{5}{{50}},\ 10.0\% $	$\frac{{21}}{{181}},\ 11.6\% $
Never	$\frac{{ < 3*}}{{219}},\ < 1.4\% $	$\frac{{ < 3*}}{{137}},\ < 2.2\% $	$\frac{{30}}{{370}},\ 8.1\% $

Values are *n* of events divided by *n* of patients, bleeding incidence.

*Value <3 censored based on the review of Findata (data permit authority for the social and health care sector in Finland) to ensure anonymity of study subjects.

BARC, Bleeding Academic Research Consortium; ARC-HBR, Academic Research Consortium for High Bleeding Risk; and HBR, high bleeding risk.

## Discussion

In this real-world STEMI-population, the ARC-HBR criteria identified patients at high bleeding risk. However, after adjusting for confounders, several individual criteria were not independent predictors of major bleeding during 1-year follow-up. Smoking status was identified as a risk factor for thoroughly adjudicated major bleeding events and current smoking fulfilled the ARC-HBR definition of a major criterion. The prevalence of current smoking was high particularly among non-HBR patients. If current smoking was considered a major ARC-HBR criterion, then 40% of non-HBR patients would have been classified as HBR resulting in 65.8% of all study patients to be regarded as HBR. Thus, recognizing smoking as a bleeding risk factor identified a large proportion of HBR patients that would otherwise have been classified as non-HBR.

Identification of HBR patients is essential, because major bleeding complications are associated with increased mortality in ACS patients. After MI, those suffering a major bleeding complication have a five-fold increased risk of death and after an ICH the risk is almost 30-fold.^1^ There is compelling evidence that patients at HBR benefit from abbreviated DAPT treatment durations.^16^ Therefore, identification of HBR-patients is required for proper management of ACS.

The ARC-HBR criteria have successfully predicted increased bleeding risk in numerous studies.^17,18^ The present study has similar findings as the 1-year major bleeding risk of 10.1% in the HBR-group was considerably higher compared to the non HBR-group (3.5%). However, performance of the individual criteria varied. In the present study including only STEMI-patients, only 4 of the original criteria remained as bleeding predictors after adjusting for confounders (age, CKD, anaemia, and malignancy). Often the ARC-HBR criteria are only adjusted with themselves, by entering the individual criteria into a multivariable model or by evaluating them in isolation without the presence of other criteria.^17^ These approaches assume that the ARC-HBR criteria include all significant bleeding risk factors. Adjustment for possible other confounding risk factors is rarely implemented. When this has been done, only few ARC-HBR criteria remain as independent bleeding predictors and new variables of interest have been identified. For example, Nakamura et al. reported that heart failure, low body weight and presentation with ACS (vs. CCS) were significant bleeding risk factors in overall PCI population.^10^ From the ARC-HBR criteria anaemia, CKD and use of OAC remained significant after adjustment. In another study by Nicolas et al., about half of the ARC-HBR criteria were not significant bleeding predictors after adjusting for confounders.^9^ Interestingly, the results differed between CCS and MI highlighting the role of clinical presentation when assessing bleeding risk. Among MI patients, anaemia, CKD, and thrombocytopenia were significant ARC-HBR criteria. It seems that anaemia and CKD are consistently associated with increased bleeding risk, which is corroborated by our results. The present study also supports advanced age and active malignancy as risk factors for bleeding among STEMI.

OAC use is widely recognized as a bleeding risk factor. Unexpectedly, use of OAC was not associated with increased risk in our data, which is contradictory to the study by Nakamura et al.^10^ Although their study population consisted of CCS and ACS patients, clinical presentation was adjusted for. Furthermore, Nicolas et al. reported that OAC use was not associated with increased bleeding risk in patients with MI but was among CCS.^9^ Therefore, OAC could bare more weight as a bleeding risk factor in CCS patients compared to ACS. It should be noted that the results are difficult to compare due to differences in the investigated population demographics and because in both previous studies, most of the ARC-HBR criteria were either modified or not available due to restrictions in registry data. Whereas the present study used the original ARC-HBR criteria except for one slightly modified criterion.

Even though the ARC-HBR framework seems to successfully predict increased bleeding risk, variables not included in the criteria were analysed to evaluate their potential influence on bleeding risk. We showed that smoking status appears to be an independent predictor for major bleeding events after MI and that current smoking fulfils ARC-HBR prerequisites for a major bleeding criterion. Smoking has been adjusted with the ARC-HBR criteria and other possible confounders in recent studies but has not been associated with bleeding risk.^8–10^ Hazard ratios and confidence intervals for smoking were not reported in several of these studies, probably because the primary objective of these studies was not to assess smoking as a risk factor, and it was not associated with increased bleeding risk. Urban et al. reported that current smoking predicted major bleeding events after PCI in a pooled cohort of HBR patients.^11^ Majority of the study population consisted of non-MI patients. The impact of current smoking was lost when analysing only those presenting with MI. Our findings suggest that current smoking is associated with bleeding complications among patients with MI. Previous studies assessed only the impact of current smoking, without considering former smoking. Therefore, current smokers were not compared with never smokers but with mixture of never and former smokers. In the present study, former smoking was associated with increased bleeding risk. This suggests that former smokers should be considered separately. Otherwise, they may act as an unmeasured confounder, potentially obscuring the true impact of current smoking. We also accounted for competing risk of deaths, which to the best of our knowledge has not been done previously and could impact results especially because current smoking at the time of PCI increases mortality.^19^

Recently smoking was shown to predict specifically upper gastrointestinal (GI) bleeding in a large cohort of MI patients.^20^ Sarajlic and colleagues concluded that current and former smokers had increased bleeding risk. Bleeding endpoint was defined as any rehospitalization with upper GI -related ICD-10 diagnosis code as primary or secondary diagnosis. The large study population and extensive adjustment for confounders support smoking as a GI bleeding risk factor after a MI. The present study extends the evidence regarding smoking as a bleeding risk factor among MI by introducing more careful endpoint adjudication and thus, adding robustness to the observation. Furthermore, we did not restrict the bleeding endpoint to a specific site but rather focused on the severity of bleeding events. This enabled the capture of all major events, which are the most prognostically significant and are the primary targets for prevention through bleeding risk assessment.

Contradictory evidence regarding smoking as a bleeding risk factor exists in general populations. A previous study reported that smoking predicted any major bleeding and bleeding in specific sites such as GI, ICH, airway, and urinary.^21^ Former smoking was associated with any major bleeding and GI bleeding. Dose-dependent increase in bleeding risk was observed when pack-years increased as well as cigarettes smoked per day among current smokers. Although interesting, these results are not applicable to ACS patients due to the investigated population and the study did not adjust for many possible bleeding risk factors often encountered in ACS, such as the ARC-HBR criteria or DAPT treatment. Also, no endpoint adjudication was used which may introduce a degree of uncertainty regarding the true severity of bleeding events. In another prospective study with more comprehensive endpoint adjudication, smoking was not a risk factor for major GI bleeding in men.^22^ Again, due to the study population and unmeasured confounders, the findings are poorly generalizable to ACS.

Recognizing current smoking as a bleeding risk factor during clinical decision making might be impactful due to several reasons. Firstly, smoking is a risk factor for coronary artery disease and is associated with future ischaemic complications after PCI.^19^ Thus, current smokers are traditionally handled as patients with high ischaemic risk which may result in longer duration of DAPT after PCI. This study suggests that these patients are also at an increased bleeding risk, which may be useful when assessing the net benefit of DAPT in patients with STEMI. However, since the balance of ischaemic and bleeding risk associated with smoking was not assessed, further evaluation is required before any application to clinical practice.

Secondly, in the present study the prevalence of current smoking was high particularly among those classified as non-HBR based on the ARC-HBR criteria. In the non-HBR group, 40.4% were current smokers vs. 16.6% in the HBR group. Therefore, recognizing smoking as a bleeding risk factor could help to identify HBR patients who would otherwise be missed. Similar findings regarding higher prevalence of smoking among non-HBR patients have been reported globally both in randomized DAPT trials and PCI registries with real-world populations. In the TWILIGHT trial, the prevalence of current smoking was 23.8% in non-HBR and 10.4% in HBR patients.^23^ The TICO trial reports high prevalences, with over 40% of non-HBR patients being current smokers and 21% in the HBR group.^24^ Furthermore, in PCI registries, current smoking is more common among non-HBR vs. HBR in Asia, Europe, and USA (39% vs. 22%, 34% vs. 16%, and 18% vs. 8.6%, respectively).^6,8,25^

Thirdly, although current smoking was more common in the non-HBR patients, it was not rare in HBR patients. This is noteworthy because the presence of multiple risk factors cumulatively increases bleeding risk.^26^ Our data corroborate this, demonstrating a 6.4% higher BARC 3 or 5 incidence among HBR patients who were current smokers compared to never smokers. Notably, the incidence was over 10% higher among those fulfilling a single minor ARC-HBR criterion.

The mechanism of how smoking causes bleeding events is speculative. Overall, smoking could damage blood vessels through toxins or impaired nitric oxide production in endothelium.^27^ This can lead to improper endothelium-dependent relaxation of arteries,^28^ resulting in increased shear stress and potential endothelial damage. One possibility is due to obtained cerebral aneurysms since smoking seems to be strongly associated with increased risk of fatal subarachnoid haemorrhage.^29^ In the present study, almost all bleeding events originating from respiratory tract occurred among current smokers and none among never smokers. Smoking has been shown to impair wound healing in the respiratory epithelium.^30^ Smoking is a well-known risk factor for cancer and bleeding risk could be mediated by this mechanism. However, this does not seem to explain our results since we adjusted for active malignancy.

Another noteworthy finding in the present study is that increasing white blood cell count was associated with increased bleeding risk. This is in line with findings from previous STEMI and non-ST-elevation ACS cohorts, although the risk increase was only modest in our dataset.^31,32^ No difference in median white blood cell count was observed between HBR and non-HBR groups but incorporating the variable into bleeding prediction models could be useful as demonstrated by the PRECISE-DAPT score.^33^ Increase in DAPT treatment duration was not associated with bleeding risk in the present study, which is surprising since randomised DAPT-trials have demonstrated reduced bleeding incidences with shorter treatment durations.^34^ It is likely that bleeding risk assessment while choosing DAPT regimens mitigated the association of this variable with bleeding in our data since short DAPT may be prescribed to HBR patients.

### Limitations

This study has several limitations. Bleeding predictors might differ in more stable CCS patients and current smoking seems to be less prevalent among them.^5^ Adherence to medication could not be ascertained due to the retrospective nature of the study and premature cessations of antiplatelet agents could have affected the results. However, a wide range of known confounders and other variables were analysed with multivariable competing risk regression. We did not have access to causes of death for subjects who died during 2023, but this probably makes little difference, since only 2 study subjects died in 2023 and during follow-up in uncertain conditions.

Over 60% had no mention of alcohol use and they were assumed to not have excessive consumption. As a sensitivity analysis, those with no mention were excluded. This had no significant difference in results. We did not do any imputations for missing data. However, it is unlikely that this affected the results since the number of missing values was low. Blood pressure has been associated with bleeding risk, but measurements were available in VSSHP institutional database only from 2021 onwards, which is why blood pressure was excluded from the study. The length and intensity of smoking history might influence how smoking impacts bleeding risk, but we did not have them recorded in the data. Furthermore, we did not have access to reliable data regarding possible smoking cessation after the index STEMI due to underreporting. Smoking cessation after PCI improves prognosis and decreases ischaemic complications.^19^ It remains unclear whether similar benefits apply to bleeding risk. Finally, this was a single centre study with moderate sample size. As a result, the generalizability of the results to other populations needs confirmation in larger multicentre studies.

## Conclusions

According to our results, current and former smoking predict major non-access site bleeding complications after STEMI and current smoking was identified as a bleeding risk factor based on the ARC-HBR definition. The prevalence of current smoking was high, particularly among patients who did not fulfill the ARC-HBR criteria. Our results suggest that guideline recommended bleeding risk assessment may fail to identify a large proportion of patients who are at HBR. Recognizing smoking as a bleeding risk factor could help to identify more high-risk patients during clinical decision making.

## Supplementary Material

qcae104_Supplemental_Files

## Data availability

Patient level data cannot be shared publicly due to legal reasons. This study uses routinely collected health and social data for a secondary purpose (research). Finnish Act on the Secondary Use of Health and Social Data (522/2019) dictates that such data can only be released into audited secure data environments.
