# Supplementary material for: Performance of the ARC-HBR criteria in ST-elevation myocardial infarction. Significance of smoking as an additional bleeding risk factor
Source: Eur Heart J Qual Care Clin Outcomes. 2024 Nov 30;11(6):756–65. doi: 10.1093/ehjqcco/qcae104 (PMC12445688; doi:10.1093/ehjqcco/qcae104)
Supplement: qcae104_Supplemental_Files [file qcae104_supplemental_files.zip › Supplementary Tables.docx]

**Supplementary materials**

| **Supplementary Table 1**. Endpoint definitions. | 2 |
| --- | --- |
| **Supplementary Table 2**. BARC subtypes for occurred bleeding events. | 2 |
| **Supplementary Table 3**. Univariable Fine-Gray regression for 1-year BARC 3 or 5 bleeding. | 3–5 |
| **Supplementary Table 4**. Multivariable Fine-Gray regression model for 1-year  BARC 3 or 5 bleeding (excluding patients with smoking status not mentioned). | 5 |
| **Supplementary Table 5**. Multivariable Fine-Gray regression model for 1-year  BARC 3 or 5 bleeding (including only patients who received PCI).  **Supplementary Table 6.** BARC 3 or 5 bleeding sites according to smoking status. | 6  7 |

Supplementary Table 1. Endpoint definitions.

| BARC Bleeding | Definition |
| --- | --- |
| Type 3a | -Any transfusion with overt bleeding.  -Overt bleeding plus haemoglobin drop ≥ 30 to < 50 g/L (provided haemoglobin drop is related to bleeding). Haemoglobin drop should be corrected for intracurrent transfusion in which 1 U packed red blood cells or 1 U whole blood would be expected to increase haemoglobin by 10 g/L. |
| Type 3b | -Overt bleeding plus haemoglobin drop ≥ 50 g/L (provided haemoglobin drop is related to bleed). Haemoglobin drop should be corrected for intracurrent transfusion in which 1 U packed red blood cells or 1 U whole blood would be expected to increase haemoglobin by 10 g/L.  -Cardiac tamponade.  -Bleeding requiring surgical intervention for control (excluding dental/nasal/skin/haemorrhoid).  -Bleeding requiring intravenous vasoactive drugs. |
| Type 3c | -Intracranial haemorrhage (does not include microhaemorrhages or haemorrhagic transformation, does include intraspinal).  -Intraocular bleeding compromising vision. |
| Type 5a | -Probable fatal bleeding. Bleeding that is clinically suspicious as the cause of death, but the bleeding is not directly observed and there is no autopsy or confirmatory imaging. |
| Type 5b | -Definite fatal bleeding. Bleeding that is directly observed (by either clinical specimen [blood, emesis, stool, etc.] or imaging) or confirmed on autopsy. |
| Fatal bleeding is bleeding that directly causes death with no  other explainable cause.  BARC, bleeding academic research consortium. | |

Supplementary Table 2. BARC subtypes for occurred bleeding events.

| Subclass | n | % Overall | % of bleeding events |
| --- | --- | --- | --- |
| 3a | 36 | 2.30 | 36.7 |
| 3b | 38 | 2.43 | 38.8 |
| 3c | 13 | 0.83 | 13.3 |
| 5 | 11 | 0.70 | 11.2 |
| BARC, bleeding academic research consortium. | | | |

Supplementary Table 3. Univariable Fine-Gray regression for 1-year BARC 3 or 5 bleeding.

| Variable | HR | 95% CI | p-value | Missing values, n (%) |
| --- | --- | --- | --- | --- |
| Clinical characteristics |  |  |  |  |
|  |  |  |  |  |
| Female sex | 1.16 | 0.77–1.77 | 0.480 | 0 (0) |
| Age | 1.03 | 1.01–1.04 | <0.001 | 0 (0) |
| Smoking | 1.26 | 1.01–1.56 | 0.038 | 0 (0) |
| Current vs never | 1.58 | 0.98–2.53 | 0.060 | 0 (0) |
| Former vs never | 1.86 | 1.14–3.04 | 0.013 | 0 (0) |
| Known excessive alcohol consumption (not mentioned = non-excessive) | 1.26 | 0.63–2.5 | 0.520 | 0 (0) |
| Excessive alcohol consumption (not mentioned = excluded) | 0.97 | 0.46–2.03 | 0.930 | Not mentioned 991 (63.4) |
| LVEF < 35 | 1.26 | 0.75–2.11 | 0.380 | 0 (0) |
| Hypertension | 1.41 | 0.93–2.12 | 0.110 | 0 (0) |
| Hypercholesterolemia | 0.73 | 0.49–1.09 | 0.120 | 0 (0) |
| Diabetes | 1.55 | 1.01–2.37 | 0.046 | 0 (0) |
| Atrial fibrillation | 2.69 | 1.75–4.13 | <0.001 | 0 (0) |
| Heart failure | 1.66 | 0.84–3.28 | 0.140 | 0 (0) |
| Prior CAD | 1.32 | 0.89–1.96 | 0.170 | 0 (0) |
| Prior MI | 1.13 | 0.66–1.96 | 0.650 | 0 (0) |
| Prior PCI | 1.09 | 0.61–1.96 | 0.760 | 0 (0) |
| Prior CABG | 1.82 | 0.76–4.39 | 0.180 | 0 (0) |
| Peripheral artery disease | 2.74 | 1.38–5.47 | 0.004 | 0 (0) |
| Prior stroke | 0.85 | 0.37–1.94 | 0.700 | 0 (0) |
| Prior ICH | 1.34 | 0.43–4.15 | 0.620 | 0 (0) |
| Laboratory values |  |  |  |  |
|  |  |  |  |  |
| Haemoglobin, g/L | 0.98 | 0.97–0.99 | <0.001 | 13 (0.83) |
| Thrombocytes, x10^9^/L | 1.0 | 1.00–1.00 | 0.580 | 14 (0.90) |
| White blood cell count, x10^9^/L | 1.04 | 1.01–1.07 | 0.017 | 14 (0.90) |
| Creatinine, mmol/L | 1.0 | 1.00–1.01 | <0.001 | 16 (1.0) |
| GFR, ml/min | 0.98 | 0.97–0.99 | <0.001 | 16 (1.0) |
| HbA1c, mmol/mol | 1.01 | 0.99–1.02 | 0.410 | 313 (20.0) |
| Medication at discharge |  |  |  |  |
|  |  |  |  |  |
| ASA | 0.70 | 0.41–1.19 | 0.190 | 0 (0) |
| Clopidogrel | 1.59 | 1.05–2.41 | 0.027 | 0 (0) |
| Ticagrelor | 0.69 | 0.46–1.03 | 0.067 | 0 (0) |
| Prasugrel | Not computable | - | - | 0 (0) |
| DAPT | 0.77 | 0.48–1.25 | 0.290 | 0 (0) |
| DAPT with ticagrelor/prasugrel | 0.62 | 0.42–0.92 | 0.016 | 0 (0) |
| DAPT duration (no DAPT as reference, < 3 months, 3-5.9 months, 6-9 months, 12 months) | 0.89 | 0.79–0.99 | 0.035 | 0 (0) |
| VKA | 0.90 | 0.29–2.84 | 0.860 | 0 (0) |
| DOAC | 2.10 | 1.27–3.47 | 0.038 | 0 (0) |
| TAT | 1.95 | 0.98–3.89 | 0.058 | 0 (0) |
| NSAID | Not computable | - | - | 0 (0) |
| Corticosteroid | 2.28 | 1.06–4.90 | 0.035 | 0 (0) |
| PPI | 1.94 | 1.31–2.89 | 0.001 | 0 (0) |
| Management |  |  |  |  |
|  |  |  |  |  |
| Primary PCI | 0.93 | 0.52–1.68 | 0.810 | 0 (0) |
| Delayed PCI (> 24 hours after symptom onset) | 1.39 | 0.44–4.38 | 0.570 | 0 (0) |
| Fibrinolysis | Not computable | - | - | 0 (0) |
| Rescue PCI | Not computable | - | - | 0 (0) |
| CABG | 0.61 | 0.08–4.55 | 0.630 | 0 (0) |
| Angiography without revascularization | 0.92 | 0.22–3.84 | 0.910 | 0 (0) |
| Non-invasive | 1.18 | 0.54–2.55 | 0.680 | 0 (0) |
| ARC-HBR |  |  |  |  |
|  |  |  |  |  |
| ARC-HBR fulfilled | 3.01 | 1.97–4.61 | <0.001 | 16 (1.0) |
| Minor criteria |  |  |  |  |
|  |  |  |  |  |
| Age ≥ 75 years | 2.18 | 1.47–3.24 | <0.001 | 0 (0) |
| Moderate CKD (eGFR 30-59.99 ml/min) | 2.18 | 1.44–3.29 | <0.001 | 16 (1.0) |
| Mild anaemia (Haemoglobin 110-129 g/L for men and 110-119 g/L for women) | 1.45 | 0.91–2.3 | 0.120 | 13 (0.83) |
| Prior spontaneous bleeding (within past 6-12 months) * | 1.79 | 0.46–6.96 | 0.400 | 0 (0) |
| Use of NSAID/steroid | 2.1 | 0.98–4.53 | 0.058 | 0 (0) |
| Prior stroke | 1.25 | 0.58–2.7 | 0.560 | 0 (0) |
| Major criteria |  |  |  |  |
|  |  |  |  |  |
| OAC use | 1.83 | 1.14–2.94 | 0.013 | 0 (0) |
| Severe or end-stage CKD (eGFR < 30 ml/min) | 1.85 | 0.91–3.78 | 0.092 | 16 (1.0) |
| Moderate or severe anaemia (Haemoglobin < 110 g/L) | 2.84 | 1.72–4.69 | <0.001 | 13 (0.83) |
| Prior spontaneous bleeding (within the past 6 months) * | 4.53 | 2.04–10 | <0.001 | 0 (0) |
| Platelet count < 100x10^9^/L | 1 | 0.14–7.37 | 1 | 14 (0.90) |
| Bleeding diathesis | 0.95 | 0.35–2.6 | 0.930 | 0 (0) |
| Liver cirrhosis | 3.46 | 0.49–24.5 | 0.210 | 0 (0) |
| Active malignancy | 4.21 | 2.35–7.56 | <0.001 | 0 (0) |
| Prior ICH/Stroke | 1.14 | 0.37–3.54 | 0.830 | 0 (0) |
| Nondeferrable surgery on DAPT | 3.65 | 0.47–28.6 | 0.220 | 0 (0) |
| Recent major surgery or trauma | Not computable | - | - | 0 (0) |
| * Requiring hospitalization or transfusion.  BARC, Bleeding Academic Research Consortium; HR, hazard ratio; CI, confidence interval; LVEF < 35, left ventricular ejection fraction < 35% at any time during index-hospitalization; CAD, coronary artery disease; MI, myocardial infarction; PCI, percutaneous coronary intervention; CABG, coronary artery bypass grafting; ICH, intracranial haemorrhage; CKD, chronic kidney disease; eGFR, estimated glomerular filtration rate (CKD-EPI formula); HbA1c, blood glycated haemoglobin; ASA, acetylsalicylic acid; DAPT, dual antiplatelet therapy; DAPT duration, prescribed duration of DAPT; VKA, vitamin K antagonist; DOAC, direct oral anticoagulant; TAT, triple antithrombotic therapy (ASA + P2Y12 receptor inhibitor + anticoagulant); NSAID, nonsteroidal anti-inflammatory drug; PPI, proton pump inhibitor; ARC-HBR, Academic Research Consortium for High Bleeding Risk; OAC, oral anticoagulant. | | | | |

Supplementary Table 4. Multivariable Fine-Gray regression model for 1-year BARC 3 or 5 bleeding (excluding patients with smoking status not mentioned).

| Variable | HR | 95% CI | p-value |
| --- | --- | --- | --- |
| Age ≥ 75 years* | 2.36 | 1.36–4.10 | 0.002 |
| OAC* | 1.57 | 0.78–3.15 | 0.200 |
| GFR 30-59.99 ml/min* | 1.62 | 0.98–2.67 | 0.059 |
| Haemoglobin < 110 g/L* | 1.84 | 0.94–3.61 | 0.075 |
| Prior bleeding (major criterion)* | 2.06 | 0.72–5.88 | 0.180 |
| Active malignancy* | 3.22 | 1.58–6.55 | 0.001 |
| Smoking | - | - | <0.001 |
| Current§ | 2.79 | 1.48–5.27 | 0.002 |
| Former# | 1.83 | 1.07–3.14 | 0.028 |
| White blood cell count (1 x 10^9^/L) | 1.04 | 1.01–1.07 | 0.012 |
| DAPT-duration$ | - | - | 0.430 |
| PPI | 1.34 | 0.87–2.08 | 0.190 |
| *ARC-HBR criterion  §Former smoking excluded (current vs. never)  #Current smoking excluded (former vs. never)  $Categories: no DAPT (reference), < 3 months, 3-5.9 months, 6-9 months, 12 months. HR and CI for category comparisons not provided because the variable was not significant.  In the model: Individual significant (Univariable Fine-Gray p <0.05) ARC-HBR criteria and other significant variables. If both major and minor criterion of the same variable was significant, major criterion was included.  BARC, Bleeding Academic Research Consortium; HR, hazard ratio; CI, confidence interval; OAC, oral anticoagulant; GFR, estimated glomerular filtration rate (CKD-EPI formula); Active malignancy, diagnosis within 12 months prior to index hospitalization or ongoing treatment. DAPT-duration | | | |

Supplementary Table 5. Multivariable Fine-Gray regression model for 1-year BARC 3 or 5 bleeding (including only patients who received PCI).

| Variable | HR | 95% CI | p-value |
| --- | --- | --- | --- |
| Age ≥ 75 years* | 2.41 | 1.38–4.21 | 0.002 |
| OAC* | 1.72 | 0.83–3.60 | 0.150 |
| GFR 30-59.99 ml/min* | 1.53 | 0.93–2.52 | 0.094 |
| Haemoglobin < 110 g/L* | 2.65 | 1.42–4.95 | 0.002 |
| Prior bleeding (major criterion)* | 1.29 | 0.41–4.03 | 0.660 |
| Active malignancy* | 4.74 | 2.42–9.31 | <0.001 |
| Smoking | - | - | <0.001 |
| Current§ | 2.88 | 1.53–5.42 | 0.001 |
| Former# | 2.07 | 1.19–3.61 | 0.010 |
| White blood cell count (1 x 10^9^/L) | 1.04 | 1.01–1.07 | 0.009 |
| DAPT-duration$ | - | - | 0.510 |
| PPI | 1.25 | 0.80–1.95 | 0.330 |
| *ARC-HBR criterion  §Former smoking excluded (current vs. never)  #Current smoking excluded (former vs. never)  $Categories: no DAPT (reference), < 3 months, 3-5.9 months, 6-9 months, 12 months. HR and CI for category comparisons not provided because the variable was not significant.  In the model: Individual significant (Univariable Fine-Gray p <0.05) ARC-HBR criteria and other significant variables. If both major and minor criterion of the same variable was significant, major criterion was included.  BARC, Bleeding Academic Research Consortium; HR, hazard ratio; CI, confidence interval; OAC, oral anticoagulant; GFR, estimated glomerular filtration rate (CKD-EPI formula); Active malignancy, diagnosis within 12 months prior to index hospitalization or ongoing treatment. DAPT-duration | | | |

Supplementary Table 6. BARC 3 or 5 bleeding sites according to smoking status.

| Bleeding site | Current  n=473 | Former  n=356 | Never  n=735 | p-value |
| --- | --- | --- | --- | --- |
| GI, n (%) | 14 (3.0) | 19 (5.3) | 20 (2.7) | 0.067 |
| ICH, n (%) | 7 (1.5) | 4 (1.1) | 6 (0.81) | 0.553 |
| Nose/mouth, n (%) | < 3 (< 0.63)* | 0 (0.0) | 0 (0.0) | 0.143 |
| Haematuria, n (%) | < 3 (< 0.63)* | 4 (1.1) | 6 (0.82) | 0.277 |
| Pulmonary/pleural, n (%) | 6 (1.3) | < 3 (< 0.84)* | 0 (0.0) | 0.003 |
| Pericardial, n (%) | 0 (0.0) | < 3 (< 0.84)* | 0 (0.0) | 0.052 |
| Genital, non-haematuria, n (%) | < 3 (< 0.63)* | 0 (0.0) | < 3 (< 0.41)* | 1.000 |
| Other, n (%) | 3 (0.63) | 0 (0.0) | < 3 (< 0.41)* | 0.201 |
| Values are n of events (absolute incidence).  *Value < 3 censored based on the review of Findata (data permit authority for the social and health care sector in Finland) to ensure anonymity of study subjects.  BARC, bleeding academic research consortium; GI, gastrointestinal; ICH, intracranial haemorrhage. | | | | |
